# Biological Materials: The Next Frontier for Cell-Free Synthetic Biology

**DOI:** 10.3389/fbioe.2020.00399

**Published:** 2020-05-12

**Authors:** Richard J. R. Kelwick, Alexander J. Webb, Paul S. Freemont

**Affiliations:** ^1^Section of Structural and Synthetic Biology, Department of Infectious Disease, Imperial College London, London, United Kingdom; ^2^The London Biofoundry, Imperial College Translation & Innovation Hub, London, United Kingdom; ^3^UK Dementia Research Institute Care Research and Technology Centre, Imperial College London, London, United Kingdom

**Keywords:** cell-free synthetic biology, biological materials, biomaterials, biomimetics, metabolic engineering

## Abstract

Advancements in cell-free synthetic biology are enabling innovations in sustainable biomanufacturing, that may ultimately shift the global manufacturing paradigm toward localized and ecologically harmonized production processes. Cell-free synthetic biology strategies have been developed for the bioproduction of fine chemicals, biofuels and biological materials. Cell-free workflows typically utilize combinations of purified enzymes, cell extracts for biotransformation or cell-free protein synthesis reactions, to assemble and characterize biosynthetic pathways. Importantly, cell-free reactions can combine the advantages of chemical engineering with metabolic engineering, through the direct addition of co-factors, substrates and chemicals –including those that are cytotoxic. Cell-free synthetic biology is also amenable to automatable design cycles through which an array of biological materials and their underpinning biosynthetic pathways can be tested and optimized in parallel. Whilst challenges still remain, recent convergences between the materials sciences and these advancements in cell-free synthetic biology enable new frontiers for materials research.

## Introduction

We live in a material world (Callén Moreu and López Gómez, 2019). There are more than five trillion plastic pieces in the world’s oceans (Eriksen et al., 2014), an estimated 15 billion trees are cut down each year (Crowther et al., 2015) and global natural fiber production is estimated to have already exceeded 30 million tons per annum (Townsend and Sette, 2016). Clearly, the mass production, processing and disposal of an array of materials has significantly shaped the global ecosystem. Whilst we continue to approach irreversible climate change (Lenton et al., 2019), it is of increasing importance that positive advances in the materials sciences are balanced against any negative environmental consequences that relate to material consumption. Arguably, these challenges warrant significant shifts in the manufacturing paradigm, from global mass production to local, sustainable and personalized manufacturing strategies (Hu, 2013; Stock and Seliger, 2016; Kleer and Piller, 2019). Likewise, instead of the chemical industries, the next generation of materials may come through developments in synthetic biology, sustainable biotechnology and the burgeoning bioeconomy (Le Feuvre and Scrutton, 2018; Philp, 2018; Freemont, 2019; French, 2019).

The field of synthetic biology has emerged over the last twenty years into a highly dynamic community of interdisciplinary researchers and societal stakeholders, that are working toward the responsible development and implementation of cutting-edge biotechnologies (Cameron et al., 2014; El Karoui et al., 2019; Lai et al., 2019). Synthetic biology combines knowledge across numerous scientific disciplines including molecular biology, biochemistry, biophysics and the social sciences, whilst also integrating them within an engineering design framework (Anderson et al., 2019; Trump et al., 2019). Ultimately, the synthetic biology approach is geared toward the rational engineering or repurposing of biological parts, devices and systems into solutions that can help address societal challenges (Ausländer et al., 2017). Synthetic biologists envision a broad application space for the field and anticipate positive societal impacts in medical technologies (Pardee, 2018; Hicks et al., 2020) (e.g., biosensors and therapeutics), food security and sustainable energy (Russo et al., 2019; Roell and Zurbriggen, 2020) (e.g., biofuels and synthetic photosynthesis), bioremediation (Dvořák et al., 2017; Wan et al., 2019) (e.g., pollution monitoring and sequestration), education (Kelwick et al., 2015a; Huang et al., 2018; Dy et al., 2019) (e.g., iGEM competition) and biomanufacturing (Le Feuvre and Scrutton, 2018; Choi K.R. et al., 2019; Gilbert and Ellis, 2019; Roberts et al., 2019) (e.g., fine chemicals and materials production).

However, biological systems are highly complex and initial attempts at engineering biological systems to fulfill specific application goals, are often only partially successful. To help overcome these challenges, synthetic biology employs the concept of the design-cycle, through which biotechnologies are iteratively designed, built and tested (Arpino et al., 2013; Church et al., 2014; Kelwick et al., 2014). Learning how to improve a biological system may require multiple attempts, which could be made easier by more rapid and systematic workflows. One potential solution is to utilize cell-free synthetic biology for rapid prototyping (Moore et al., 2017b; Kelwick et al., 2018). Typically, cell-free reactions make use of isolated cellular components and machinery (e.g., ribosomes and recombinant proteins), rather than live whole-cells. Whilst, some cell-free reaction components may be cell-derived (e.g., cell extracts), once prepared, cell-free workflows can be completed within hours (Chappell et al., 2013). In contrast, typical whole-cell experiments may involve several days or weeks of delays that are associated with plasmid cloning, transformation and cell growth (Sun et al., 2014). Furthermore, cell-free reactions are accessible and can combine the advantages of chemical engineering with metabolic engineering, through the direct addition of enzyme co-factors, substrates and chemicals – including those that are cytotoxic (Dudley et al., 2015; Karim and Jewett, 2016; Kay and Jewett, 2020). These advantages are increasingly being exploited for cell-free applications including biopart prototyping, cell-free metabolic engineering, medical or environmental biosensors and on-demand therapeutics production (Kightlinger et al., 2019; Silverman et al., 2019). Based upon an expanding repertoire of examples in the literature, we envision that biological materials and bio-functionalized smart materials are the next frontier for cell-free synthetic biology (Table 1). To this end, this review will introduce key concepts and recent developments in cell-free synthetic biology, with a focus on examples relevant to the materials sciences. Examples will be given of industrially and societally important biological materials that have been generated using cell-free synthetic biology. Cell-free synthetic biology can also be utilized to bio-functionalize materials, which may further enable the emergence of new types of smart materials. This review will also explore future trends and challenges in cell-free synthetic biology and speculate on their potential impact on biological materials of the future.

**TABLE 1:** Cell-free strategies for biological material biomanufacturing or material bio-functionalization.

**Cell-free reaction format**	**Material**	**Application**	**References**
Recombinant enzymes	Polyhydroxyalkanoates (PHAs)	Biopolymer production	Thomson et al., 2009; Han et al., 2011; Tomizawa et al., 2012; Opgenorth et al., 2016
	Lactic acid	Platform material for polymer production	Kopp et al., 2019
Cell extract biotransformation	Bio-cellulose	Bio-cellulose production	Ullah et al., 2015
	Chitin	Chitin synthesis	Jaworski et al., 1963
	Poly-3-hydroxybutyrate [P(3HB)]	Optimizing PHAs biopolymer production	Kelwick et al., 2018
	Gold nanoparticles (AuNPs)	Medical and industrial	Chauhan et al., 2011; Krishnan et al., 2016
	Silver nanoparticles (AgNPs)	Nanobiotechnology, therapeutic development	Costa Silva et al., 2017
Cell-free protein synthesis	Bacteriophages	*De novo* synthesis and phage engineering	Garamella et al., 2016; Rustad et al., 2018
	Chitin	Chitinase expression	Endoh et al., 2006
	Clay microgels	Protein production	Jiao et al., 2018
	DNA hydrogels/Protein-producing gels (P-gel)	Protein production	Park et al., 2009a; Ruiz et al., 2012
	Elastin-like polypeptides (ELPs)	Biopolymer with non-canonical amino acids	Martin et al., 2018
	Extracellular vesicles (EVs)	Therapeutics/EV biogenesis research	Shurtleff et al., 2016; García-Manrique et al., 2018
	Freeze-dried pellets	*In vitro* diagnostics or therapeutic production	Pardee et al., 2016b; Salehi et al., 2016, 2017
	Liposomes and nanodiscs	Membrane protein production, drug discovery or protocell production	Garamella et al., 2016; Rues et al., 2016; Shinoda et al., 2016; Contreras-Llano and Tan, 2018; Gessesse et al., 2018; Dubuc et al., 2019; Shelby et al., 2019
	Microfluidic devices (various)	Antibody development and protein microarrays	Kilb et al., 2014; Georgi et al., 2016; Contreras-Llano and Tan, 2018
	Microparticles/nanoparticles	On-demand functional biomaterials/therapeutics	Lim et al., 2009; Benítez-Mateos et al., 2018
	Paper	*In vitro* diagnostics	Pardee et al., 2014, 2016a; Duyen et al., 2017; Gräwe et al., 2019; Thavarajah et al., 2020
	PEG hydrogels	Education	Huang et al., 2018
	Poly-3-hydroxybutyrate (P(3HB))	Polyhydroxyalkanoates (PHAs) biosynthetic operon prototyping	Kelwick et al., 2018
	Protein biologics	Cancer therapeutics, protein therapeutics	Zawada et al., 2011; Sullivan et al., 2016; Salehi et al., 2017; Kightlinger et al., 2019
	Silk fibroin	Silk fibroin production	Greene et al., 1975; Lizardi et al., 1979

## Cell-Free Synthetic Biology Reaction Formats and Strategies

**FIGURE 1:**
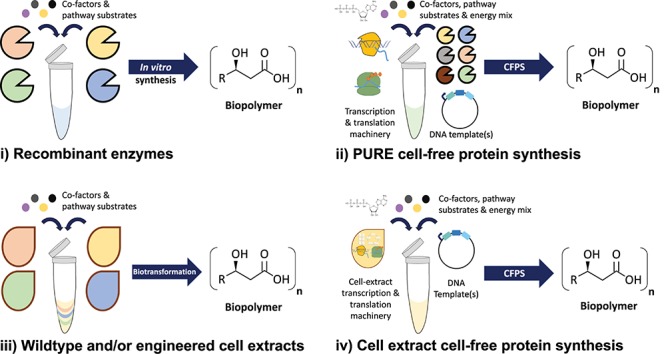
Cell-free synthetic biology reaction formats and strategies. **(i)** Recombinant enzymes can be mixed together along with enzyme co-factors and substrates to form biosynthetic pathways. **(ii)** The PURE cell-free protein synthesis system utilizes reconstituted *Escherichia coli* transcription and translation machinery, DNA templates, purified enzymes and other factors. **(iii)** Cell extracts from lysed wildtype or engineered cells can be mixed together along with enzyme co-factors and substrates to form biosynthetic pathways. **(iv)** Cell extract-based cell-free protein synthesis reactions utilize the transcription and translation machinery within cell lysates, along with exogenously added energy mix components (e.g., amino acids) and DNA templates for *in vitro* protein production.

Cell-free synthetic biology is a broad term that encompasses many different *in vitro* biotechnologies. Broadly, the term cell-free synthetic biology refers to different methods and technologies for engineering or using biological processes outside of a cell. For example, cell-free protein synthesis reactions enable the production of proteins within biochemical reactions. Thus, cell-free reactions typically make use of isolated cellular components (e.g., recombinant proteins) and/or cell extracts, rather than live whole-cells. In the context of this review four commonly used cell-free reaction formats will be discussed (Figure 1). We describe these cell-free reaction formats as either (i) recombinant enzyme-based, (ii) protein synthesis using recombinant elements (PURE)-based cell-free protein synthesis, (iii) wildtype and/or engineered cell extract biotransformation or (iv) cell extract-based cell-free protein synthesis.

Recombinant enzyme-based reaction formats utilize purified enzymes, along with any required co-factors and pathway substrates, to produce fine chemicals, polymer monomers or other molecules of interest. The PURE-based cell-free protein synthesis format reconstitutes the transcription and translation machinery from *Escherichia coli* using purified histidine (His)-tagged proteins (Shimizu et al., 2001, 2005). In this reaction format, the exact components are known, including the co-factors, substrates and energy mixes. Since PURE reaction components are known they can be standardized and rationally optimized. However, PURE cell-free reactions typically produce lower protein yields than cell-free protein synthesis reactions that use *E. coli* extracts (Shimizu et al., 2005). The third cell-free reaction format uses cell extracts from lysed wildtype and/or engineered cells, which can be mixed together along with relevant required enzyme co-factors and substrates to form multicomponent biosynthetic pathways. Finally, the last format, cell extract-based cell-free protein synthesis (CFPS), uses the transcription and translation machinery from lysed cells, along with added co-factors and energy mixes to produce proteins *in vitro*. Cellular extract-based cell-free reactions use the host cells native transcription and translation machinery as well as other metabolic components, including energy providing enzymes such as those involved in glycolysis and the Krebs cycle, which are released when the cells are lysed either mechanically (French pressure cell press), by sonication or osmotically (Gregorio et al., 2019). These reactions were originally developed as experimental tools to enable the fundamental understanding of aspects of cellular biochemistry, molecular biology and *in vitro* production of various proteins of interest (Gagoski et al., 2016). A range of different host cells have been used to develop these reactions, including bacteria such as *Bacillus subtilis* (Kelwick et al., 2016), *Streptomyces venezuelae* (Moore et al., 2017a; Li et al., 2018) and *E. coli* (Sun et al., 2013) as well as insect (Ezure et al., 2006), wheat germ (Harbers, 2014), yeast (Hodgman and Jewett, 2013; Aw and Polizzi, 2019), protozoans such as *Leishmania tarentolae* (Mureev et al., 2009; Kovtun et al., 2010, 2011) and mammalian cells (Weber et al., 1975; Martin et al., 2017).

It is important to note that these different cell-free reaction formats are not mutually exclusive and can be combined together. Recombinant enzymes or small molecule substrates can also be added into cell-free protein synthesis reactions to complete biosynthetic pathways, or to use exogenous chemistries within the reaction. It is this flexibility that we envision being particularly useful in terms of exploring how cell-free reactions could create novel types of biological materials or bio-functionalized smart materials. In the following sections we discuss exemplars where cell-free synthetic reactions have been utilized to prototype, manufacture or bio-functionalize, biological materials (Table 1).

## Cell-Free Strategies for Sustainable Materials Biomanufacturing

Living cells and organisms have evolved highly complex enzymes and metabolic processes that generate extremely diverse biochemistries. Exploring these natural biochemistries may lead to important foundational advances in our understanding of natural product synthesis. Foundational discoveries in functional genomics, cellular metabolism and natural product synthesis are also important, because they might inspire novel biosynthetic pathway designs for biological materials production. In synthetic biology, cell-free metabolic engineering (CF-ME) approaches can reconstitute entire biosynthetic pathways using either cell extracts from diverse species, engineered cells and/or cell-free synthesized recombinant enzymes (Karim and Jewett, 2018; Martin et al., 2018; Yim et al., 2019; Bowie et al., 2020) (Figure 1). Also, cell-free protein synthesis and cell extract biotransformation reactions can be combined to create more complex cell-free reactions (Karim and Jewett, 2018; Kelwick et al., 2018). Another important advantage in using cell-free approaches is that pathway reaction bottlenecks can be identified, through the direct addition of the required recombinant enzymes, enzyme co-factors or chemical substrates needed for each stage of a biosynthetic pathway (Dudley et al., 2015). Increasingly sophisticated combinatorial CF-ME strategies, together with high-throughput automation, deep data omics and design of experiments (DoE) approaches to cell-free reaction optimization have been deployed (Caschera et al., 2018; Jiang et al., 2018; Dopp et al., 2019). These advancements have considerably improved the feasibility of refactoring and optimizing fine chemical or natural product biosynthetic pathways within short timeframes (Dudley et al., 2015; Korman et al., 2017; Moore et al., 2017a; Wilding et al., 2018).

Cell-free synthetic biology approaches have also been directed toward the *de novo* bioproduction of biological materials, including biopolymers or their monomers, cellulosic materials and nanoparticles (Table 1). However, the maximum cell-free bioproduction yields or reaction efficiencies of several reported materials were generally low or unspecified. Examples of cell-free produced materials and their reported maximum production yields and reaction efficiencies include bio-cellulose (3.726 ± 0.05 g/L; 57.68%) (Ullah et al., 2015), chitin (yields not stated) (Jaworski et al., 1963; Endoh et al., 2006), lactic acid (6.6 ± 0.1 mM; 47.4 ± 3.9%) (Kopp et al., 2019), gold nanoparticles (yields not stated) (Chauhan et al., 2011; Krishnan et al., 2016), (*R*)-3-hydroxybutyrate-CoA (32.87 ± 6.58 μM) (Kelwick et al., 2018), silver nanoparticles (yields not specified) (Costa Silva et al., 2017) and silk fibroin (yields not specified) (Greene et al., 1975; Lizardi et al., 1979). Poor cell-free production yields and efficiencies can be due to a variety of factors including rapid depletion of reaction energy mix components (e.g., ATP, amino acids), the formation of inhibitory waste products (e.g., inorganic phosphates) or unwanted side reactions that divert reaction fluxes away from desirable pathways (Caschera and Noireaux, 2014). Because of these limitations, cell-free synthetic biology may not be an ideal production method for some biological materials. Nevertheless, whilst actual cell-free material production yields can be relatively low, these approaches are still beneficial for prototyping different biosynthetic pathways, substrates or reaction conditions to boost both *in vitro* and whole-cell production yields. An exemplar is the use of cell-free assays to characterize polyhydroxyalkanoates (PHAs) biosynthetic pathways from *phaCAB* operons that also enhanced *in vivo* PHAs production (Kelwick et al., 2018). Furthermore, the same study also demonstrated that the cell-extract biotransformation of whey permeate into 3-hydroxybutyrate (3HB), could be simultaneously coupled with the cell-free protein synthesis of a potential Acetyl-CoA recycling enzyme (Kelwick et al., 2018). Thus, highlighting that combinatorial cell-free reaction formats can be a useful strategy for bioplastic pathway prototyping and optimization.

Interestingly, in some cases, cell-free bioproduction may actually be a more desirable manufacturing route. For instance, several *in vitro* gold or silver nanoparticle production studies reported desirable nanoparticle characteristics (e.g., size/zeta potential) and/or easier purification protocols within cell-free bioproduction reactions than whole-cell production methods (Krishnan et al., 2016; Costa Silva et al., 2017). Cell-free bioproduction can also be carried out at industrially relevant scales, as illustrated by Sutro biopharma who have developed a highly scalable good manufacturing practices (GMP)-compliant cell-free protein synthesis platform, for producing therapeutic proteins within 100 L bioreactor reaction volumes (Zawada et al., 2011). For cell-free materials production, a highly efficient synthetic biochemistry module was developed to convert glucose into bio-based chemicals, including the PHA bioplastic monomer polyhydroxybutyrate (PHB) (Opgenorth et al., 2016). To achieve this, purified recombinant enzymes were used to reconstitute core elements of the pentose, bifido, glycolysis and PHB pathways (Opgenorth et al., 2016). Cell-free PHB production yields (40 g/L) and efficiencies (90%) were impressive and are promisingly close to industrially attractive scales (Opgenorth et al., 2016). These improvements in PHB production are also welcome since PHB, as well as other PHAs biopolymers, are biodegradable and can potentially be used as ‘drop-in’ replacements for oil-derived plastics (e.g., food packaging) or as biomaterials for tissue engineering (Choi S.Y. et al., 2019; Tarrahi et al., 2020). PHAs are also an interesting example because of their industrial importance and the diversity of cell-free strategies that have been applied to PHAs research (Table 1). Building upon these examples, CFME approaches could be used to explore a greater diversity of PHAs biopolymers given that PHA biopolymers can be composed of a variety of different wildtype and/or synthetic monomers (∼160 different monomers exist) to create complex co-polymers, with an array of material characteristics (Choi S.Y. et al., 2019). Future cell-free synthetic biology explorations of PHAs are likely to unlock novel PHAs biopolymers with unique characteristics (Chen and Hajnal, 2015) and therefore, accelerate bioplastic materials development.

**FIGURE 2:**
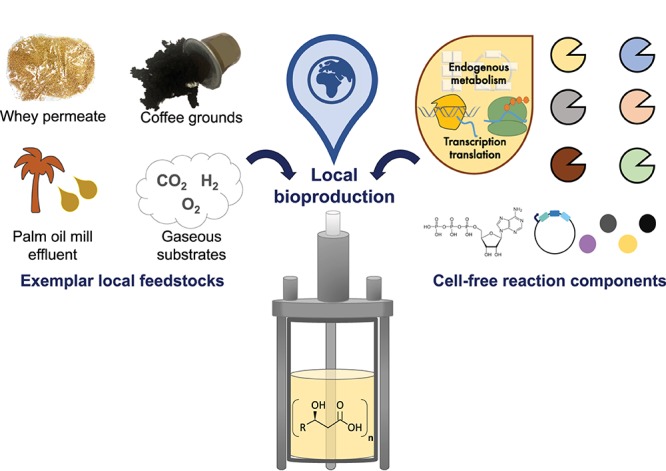
Sustainable cell-free biomanufacturing of biological materials. Schematic depicts the local, on-demand cell-free mediated, biomanufacturing of biological materials. Local feedstocks can potentially be utilized as replacements for expensive reaction energy mix components, or to provide the enzymatic co-factors and biosynthetic pathway substrates that are required to produce biological materials of interest.

Microbial (*in vivo*) PHAs production has been commercially manufactured at industrial scales over the last several decades. Unfortunately, the commercial impact of PHA-based bioplastics has been historically prohibited by their higher production costs than oil-derived plastics (Chen et al., 2020). However, more efficient PHAs production processes have been devised through the rational design of *phaCAB* biosynthetic pathways (Hiroe et al., 2012; Kelwick et al., 2015b; Li et al., 2016; Tao et al., 2017; Zhang X. et al., 2019), key metabolite recycling processes (e.g., Acetyl-CoA) (Matsumoto et al., 2013; Beckers et al., 2016), alternative microbial production hosts (e.g., *Halomonas* sp., Tan et al., 2011) and the use of industrially sourced, low-cost feedstocks (e.g., whey permeate) (Wong and Lee, 1998; Ahn et al., 2000; Kim, 2000; Nikel et al., 2006; Cui et al., 2016; Nielsen et al., 2017). Interestingly, several of these microbial PHAs production strategies are also compatible with cell-free synthetic biology reactions. In particular, using locally sourced, low-cost feedstocks (e.g., whey permeate) may help to make cell-extract based PHAs production more economically viable (Kelwick et al., 2018). A similar approach has already been piloted for cell-free lactic acid production from spent coffee grounds (Kopp et al., 2019) and could become a generalized strategy for sustainable cell-free materials bioproduction (Figure 2). We would argue that combining cell-free extracts with local feedstocks enables immediate access to highly diverse cellular biochemistries and low-cost substrates (e.g., waste feedstocks), that could potentially be used for the sustainable biomanufacturing of a diverse array of biological materials (Yan and Fong, 2015; Le Feuvre and Scrutton, 2018). Furthermore, just as lyophilized cell-free reactions enable the on-demand production of biotherapeutics (Pardee et al., 2016b), we likewise envision that cell-free reactions might one day lead to rapid and distributed, on-demand biological materials production or bio-functionalization.

## Cell-Free Material bio-Functionalization and Biomimetics

Cell-free systems are still being applied to understand the foundational principles and molecular mechanisms of transcription and translation (Hecht et al., 2017; Borkowski et al., 2018). Unlocking these principles can also potentially identify novel strategies to enhance *in vitro* protein production, which continues to be a core application for cell-free synthetic biology (Gagoski et al., 2016; Khambhati et al., 2019). Simplified or improved cell-free methodologies (Sun et al., 2013; Kwon and Jewett, 2015; Krinsky et al., 2016; Katsura et al., 2017; Lavickova and Maerkl, 2019), coupled with lower-cost and rationally optimized cell-free reaction energy mixes (Cai et al., 2015), has enabled the implementation of *in vitro* therapeutic protein production at industrial scales (Ogonah et al., 2017; Garenne and Noireaux, 2019). Interestingly, the integration of cell-free reactions within materials can also enhance cell-free protein synthesis yields. For instance, the Luo group has developed the protein-producing gel (P-gel) platform, which integrates protein-producing cell-free reactions within enzyme-catalyzed, 3D DNA hydrogel matrices (Park et al., 2009a). P-gels have been used to produce a panel of model proteins including green fluorescent protein (GFP), chloramphenicol acetyltransferase (CAT) and *Renilla reniformis* luciferase protein (Park et al., 2009a, b). Optimized P-gels reportedly generated between 1.87 and 5 mg/ml^–1^ of functional luciferase protein, which was significantly higher (>90-fold) than comparative, standard liquid-format cell-free reactions (Park et al., 2009a, b). However, the authors compared P-gels against linear DNA templates within standard, liquid-format cell-free reactions. In comparison to plasmid DNA, linear DNA fragments can be relatively unstable in cell-free reactions and this instability can result in relatively low cell-free protein production yields (Chappell et al., 2013; Sun et al., 2014). Nevertheless, the P-gel platform is potentially a flexible and versatile platform for cell-free protein production. Furthermore, P-gel droplets can also be rapidly fabricated using microfluidics – thus making scale-up more feasible (Ruiz et al., 2012). In a separate study, a microfluidic fabrication strategy has been developed to embed cell-free protein synthesis reactions within clay microgels that could produce >1 mg/ml of GFP protein (Jiao et al., 2018). Importantly, P-gels and clay microgels illustrate that the cell-free functionalization of materials can lead to novel approaches for protein biomanufacturing.

Combining cell-free synthetic biology with materials can also be important for other applications. For example, by combining the cell-free protein synthesis of membrane proteins with liposomes or nanodiscs can potentially facilitate the production and stable integration of membrane proteins within lipid bilayers – thus enabling structure-function studies or drug discovery applications (Shinoda et al., 2016; Gessesse et al., 2018; Shelby et al., 2019). In a separate study, the Schekman group recreated aspects of exosome biogenesis *in vitro*, by using cell-free reactions to examine exosome membrane protein topology and exosome-associated miRNA sorting (Shurtleff et al., 2016). These cell-free approaches also enable directed membrane functionalization - potentially leading to rationally engineered protocells and designer exosomes. Combining cell-free synthetic biology with materials has led to the bottom-up engineering of biomimetics, including synthetic protocells (Shin and Noireaux, 2012; Huang et al., 2013; Garamella et al., 2016; Dubuc et al., 2019; Yue et al., 2019), entire phages (Rustad et al., 2018) and more recently, a partially self-replicating *in vitro* translation system was reported that functions by activating a 116 kb genome and is an important step toward a living, synthetic cell (Libicher et al., 2020).

## Cell-Free Synthetic Biology Enabled Smart Materials

**FIGURE 3:**
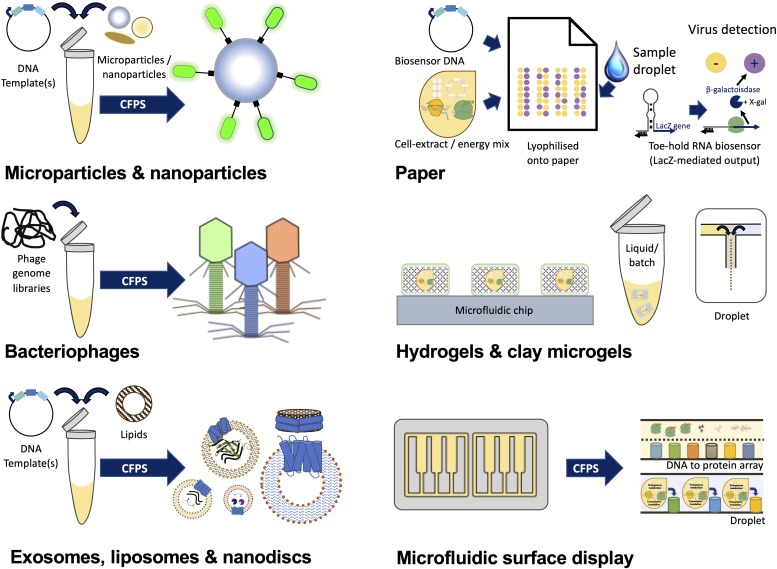
Cell-free synthetic biology-based material functionalization. Schematic depicts examples of materials that have been bio-functionalized using cell-free protein synthesis (CFPS) reactions.

Cell-free synthetic biology reactions also enable smart materials and biosensor applications. Cell-free synthetic biology reactions can be programmed with plasmid-encoded gene circuits, lyophilized and embedded within paper (Pardee et al., 2014, 2016a; Smith and Berkheimer, 2014; Pardee, 2018), as well as potentially other materials such P-gels or clay microgels. Cell-free paper-based biosensors can be activated, post-lyophilization, using water or liquid samples and have been shown to maintain activity even after several months of storage at room temperature (Pardee et al., 2014; Smith and Berkheimer, 2014). Once activated, these paper-based cell-free reactions enable *in vitro* biosensor applications where these cell-free reactions are programmed to generate detectable signal outputs in response to the presence of relevant molecules (e.g., Mercury) or disease biomarkers (Lee and Kim, 2019; Silverman et al., 2019). Beneficially, these cell-free smart materials also provide greater flexibilities in terms of their usage beyond the laboratory and outside in the field (Pardee et al., 2016a, b). The complexity of synthetic biology genetic circuits and the strategies used to devise them has increased significantly in recent years (Nielsen et al., 2016; Grunberg and Del Vecchio, 2020). This has resulted in the development of an array of cell-free compatible genetic circuits that incorporate a variety of different regulatory elements that are applicable to different biosensing applications. For example, cell-free compatible gene networks can be transcription-based such that the presence of a small molecule induces reporter gene expression, through binding to, and activation or repression of, a transcriptional regulator. These types of transcriptional gene circuits have been used to develop cell-free biosensors for detecting heavy metals (Gräwe et al., 2019; Gupta et al., 2019), a date-rape drug (Gräwe et al., 2019), metabolites (Voyvodic et al., 2019) and quorum sensing molecules from *Pseudomonas aeruginosa*-infected respiratory samples (Wen et al., 2017). Small molecules can also regulate transcription by binding to endogenous or engineered RNA aptamer-regions within 5′ untranslated (UTR) mRNA regions – termed riboswitches. Small molecule binding to the riboswitch facilitates the emergence of stable RNA structures that permit continued reporter gene transcription. Small molecules can regulate translation via a different mechanism, whereby binding of the small molecule to the riboswitch influences the mRNA structure such that it occludes ribosome binding and downstream translation (Nahvi et al., 2002). Utilizing these principles a cell-free biosensor was developed that exploits a riboswitch to detect environmental fluoride (Thavarajah et al., 2020). Cell-free biosensors can also utilize toeholds to detect RNAs (e.g., viral RNA). Essentially, toehold aptamers function in a similar way to riboswitches except that the presence of a complementary RNA is responsible for a conformational change in the mRNA that enables ribosome access and reporter protein translation (Figure 3). Engineered cell-free toehold biosensors can also differentiate between different Ebola (Pardee et al., 2014) and Zika (Pardee et al., 2016a) strains. Conceivably, RNA aptamer-based approaches could be adapted to detect Coronaviruses such as SARS-CoV and 2019 n-CoV (Ahn et al., 2009). Indeed, the 2020 COVID-19 pandemic might be a catalyst for the development of cell-free viral biosensors and the accompanying clinical studies that will be required for comparative testing against existing technologies (e.g., quantitative reverse transcription polymerase chain reaction, RT-qPCR) (Liu et al., 2020). Post-translational cell-free biosensors have also been developed to detect glucose. In this example, cell-free protein synthesis was used to produce fusion protein pairs that elicit changes in Fluorescence Resonance Energy Transfer (FRET) signals in response to bound glucose (Pardee et al., 2014). Furthermore, purified fusion proteins can also enable other types of smart material applications. For instance, Wagner et al. (2019) developed information-processing materials that function by using protease-based signal-amplifying cascades, that integrate both proteolytic activities and ligand-receptor sensing within its logic circuits. As an exemplar, the same authors reported on the development of smart materials, inspired by synthetic biology logic circuits, that can detect novobiocin antibiotics (Wagner et al., 2019). These, as well as many other transcriptional and translational regulatory mechanisms can be combined into highly complex cell-free executable circuit designs (Jeong et al., 2019). Therefore, it is conceivable that these studies might inspire future efforts to embed cell-free executable logic circuits within a broad array of synthetic biology-based smart materials.

## Automated Design-Cycles for Cell-Free Biological Materials

An important future trend in synthetic biology is the maturation of rational, engineering-led strategies for designing and implementing complex biological systems. Essentially, the field of synthetic biology envisions a future where model-guided, forward-engineering strategies will be routinely used to iterate design-build-test-learn cycles toward the final biotechnology production and application (Kelwick et al., 2014; Bultelle et al., 2016; Misirli et al., 2019). However, the scale of experiments needed to realize this vision may require continued advancements in synthetic biology machine-learning strategies, coupled with automation equipment (e.g., liquid handling robots) (Bultelle et al., 2016; Rajakumar et al., 2019). Indeed, automation capabilities can greatly expand the scale, sophistication and scope of synthetic biology design cycles (Kelwick et al., 2014; Carbonell et al., 2019; El Karoui et al., 2019; Hillson et al., 2019; Jessop-Fabre and Sonnenschein, 2019). Co-ordinated efforts toward improving inter-laboratory data reproducibility, standardizing experimental metrology and an emphasis on industrially scalable biotechnology are also driving the adoption of automation in synthetic biology workflows (Kelly et al., 2009; Beal et al., 2016, 2018a,b; de Lorenzo and Schmidt, 2018; Carbonell et al., 2019; Exley et al., 2019). In a broader sense, automation is also becoming more accessible through reductions in gene synthesis costs (Carlson, 2014), advancements in automated DNA assembly protocols (Kanigowska et al., 2016; Rajakumar et al., 2019; Storch et al., 2019; Walsh et al., 2019), rapid mass spectrometry of complex biological samples (Gowers et al., 2019; Miguez et al., 2019; O’Kane et al., 2019) and through the emergence of academic biofoundries (Chambers et al., 2016; Hillson et al., 2019).

**FIGURE 4:**
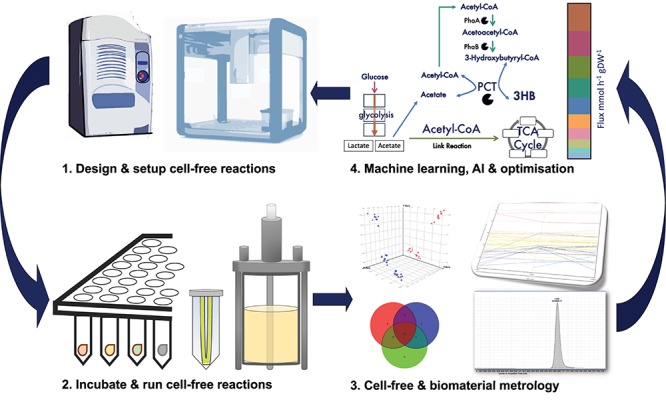
Automated cell-free design-cycles for biological materials development. Schematic depicts a cell-free design-cycle for prototyping or biomanufacturing biological materials. Firstly, cell-free reactions are designed and then setup using liquid handling robots. Secondly, these cell-free reactions are incubated in an array of reaction formats including batch or continuous feed, at a range of scales (μl to L). Thirdly, cell-free reactions and biological materials are assayed and characterized. Finally, these data inform cell-free reaction models (e.g., metabolic flux models) or material quality control benchmarks that ultimately inform the next iteration of biosynthetic pathways and cell-free reaction parameters to be tested. Sophisticated design-cycle workflows may also utilize machine learning and design-of-experiment (DOE) approaches, to rationally iterate design cycles toward the desired biological material.

Cell-free prototyping strategies can be readily integrated into design-cycles, including those applications that are intended to be functional in living cells (*in vivo*). Several studies have described comparability between DNA regulatory elements and genetic circuits tested *in vitro* (cell-free) and *in vivo* (whole-cells) across several model (e.g., *E. coli*, *Bacillus subtilis*) (Chappell et al., 2013; Sun et al., 2014; Kelwick et al., 2016) and non-model (e.g., *Bacillus megaterium, Vibrio natriegens*) (Failmezger et al., 2018; Moore et al., 2018) organisms. Thus, cell-free rapid prototyping strategies can also be applied to speed up the development of *in vivo* (whole-cell) applications. Cell-free workflows are also amenable to automation. Indeed, several studies have successfully utilized acoustic liquid handling robots to rapidly setup large-scale, low-volume (≤10 μl) prototyping cell-free reactions in a 384 well plate format (Moore et al., 2018; Kopniczky et al., 2020). Microfluidic (Swank et al., 2019), droplet array (Zhang Y. et al., 2019) or multiplex (Yim et al., 2019) strategies have also been used to enable high-throughput cell-free experiments. These approaches enable the testing of large numbers of regulatory elements or enzymes, which could potentially inform material biosynthetic pathway optimization. Conceivably, cell-free optimized material biosynthetic pathways could also be implemented within *in vivo* production strategies (e.g., microbial cell factories). These data sets also enable accompanying quantitative modeling that can potentially predict unknown model parameters, such as transcription factor binding affinities or cell-free energy utilization (Bujara and Panke, 2012; Moore et al., 2018). These data can potentially be entered into biopart data repositories (Ham et al., 2012; Bultelle et al., 2016; McLaughlin et al., 2018) for use in machine learning-enhanced design of experiments (DoE) approaches, to speed up materials development (Liu et al., 2017; Exley et al., 2019; Madsen et al., 2019). High-throughput cell-free experiments can also tease apart where cell-free reactions are fundamentally different to native intracellular environments. Molecular crowding, metabolic fluxes, co-factor regeneration rates and other biophysiochemical characteristics may all differ between cell-free reactions and *in vivo* (whole-cell) contexts. Also, cell extract processing breaks down cellular organelles and other intracellular compartments, thus perturbing native biomolecule localization. Yet, despite these potential limitations complex genetic circuits (e.g., oscillators), that are functional *in vivo*, have been prototyped using cell-free forward-engineering design cycles (Niederholtmeyer et al., 2015). This review has also explored several other examples where cell-free approaches have been used to produce or bio-functionalize materials (Table 1). It is therefore conceivable, that cell-free synthetic biology design-cycles may strengthen future efforts to accelerate the development of an array of bio-functionalized smart materials (Figure 4).

## Summary and Outlook

In an era of extreme climate events there is an increasing global consensus to collectively implement more sustainable and carbon-neutral policies (Gills and Morgan, 2019). However, there continues to be an insatiable global demand for materials and the natural resources that are used to manufacture them. These activities have, however, negatively impacted the global ecosystem through for example deforestation, industrial pollution and biodiversity destruction. To partially address some of these challenges, there is increasing support for more locally based bioeconomies underpinned by sustainable practices. Cell-free biomanufacturing could have an important role in the emerging bioeconomy by enabling biological materials to be produced locally, on-demand and more sustainably from waste feedstocks (e.g., whey permeate). Furthermore, centrally produced cell-free reaction components can be lyophilized and then distributed to different geographical locations (Pardee et al., 2016b). Once distributed, these cell-free reactions could potentially make use of highly customized DNA-encoded biosynthetic operons to produce personalized materials that suit local needs. Conceivably, these cell-free produced biological materials could be fabricated using synthetic biochemistries [e.g., unnatural amino acids (Martin et al., 2018; Des Soye et al., 2019; Gao et al., 2019) and xeno nucleic acids (Glasscock et al., 2016; Hu et al., 2019)], mixed cell extracts from diverse bacterial species (Yim et al., 2019), or *de novo* biological components [e.g., engineered ribosomes (Caschera et al., 2018; d’Aquino et al., 2020) and rationally designed proteins (Huang et al., 2016; Rolf et al., 2019)]. These synthetic components might confer cell-free produced materials with unique physical characteristics or other attributes that are not typically associated with natural fibers. Likewise, we envision that future cell-free materials will have integrated smart-features including biosensing capabilities, the ability to change material properties in response to specific stimuli or self-healing capabilities when damaged. Such capabilities may require further advancements in cell-free genetic circuits and the methods used to embed them within different materials.

A panoply of recent examples, including those discussed in this review, are indicative that biological materials are the next frontier for cell-free synthetic biology – but challenges remain. A deeper understanding of the compositions and biochemical activities of processed cell extracts and cell-free reactions are needed (Foshag et al., 2018) to mitigate cell-free extract batch variability and increase protein synthesis yields. Compounding these challenges are the need for improved cell-free metrology and cell-free biomanufacturing quality control standards, which are especially important for industrial applications (de Lorenzo and Schmidt, 2018). Equally, the acceptability of cell-free produced biological materials may need to be considered through stakeholder engagement, consumer awareness activities and through, where necessary, the establishment of specialist recycling/waste management infrastructure. The scalability of cell free reactions to industrial manufacturing is also extremely challenging. Despite these challenges, Genomatica, Greenlight Biosciences, Sutro Biopharma, Tierra Biosciences and other companies have already successfully developed scalable cell-free synthetic biology platforms that have at least pilot tested materials production (e.g., Genomatica’s cell-free platform produces polymer chemicals including 1,4-butanediol). In addition, the synthetic biology companies Bolt Threads, Spiber, Colorifix, and Ginkgo Bioworks are also working toward a future sustainable fashion industry. Bolt Threads produce spinnable recombinant spider silk in yeast and are also developing scalable biomanufacturing processes for mushroom-based leather. Spiber are also producing recombinant silk, as well as other synthetic protein materials. Both Bolt Threads and Spiber have successfully manufactured pilot batches of clothing products for a future sustainable fashion industry. Colorifix have developed a biological textile dye process that is designed to be less polluting than traditional chemical methods. Meanwhile, Ginkgo Bioworks have made significant investments into their organism-engineering foundry to support an array of manufacturing applications – including the production of materials. Ginkgo Bioworks also occasionally uses cell-free metabolic engineering strategies within its innovation pipelines.

In summary, cell-free synthetic biology is a powerful, highly customizable and promising biotechnology that is beginning to have a positive impact on the industrialization of sustainable materials production. Importantly, synthetic biology companies continue to champion interdisciplinary collaborations with designers and materials scientists as part of the development process. We envision that a continuation of these trends will result in a new frontier for sustainable cell-free materials production and the growing bioeconomy.

## Author Contributions

All authors listed have made a substantial, direct and intellectual contribution to the work, and approved it for publication.

## Conflict of Interest

The authors declare that the research was conducted in the absence of any commercial or financial relationships that could be construed as a potential conflict of interest.
